# Dr. John Cooke on the Various Species of Palsy

**Published:** 1821-03-01

**Authors:** 


*History and Method of Cure of the various Species of Palsy; being the first part of the second volume of a Treatise on Nervous Diseases.* By John Cooke, M.D. F.A.S. Fellow of the Royal College of Physicians, and late Physician to the London Hospital. Octavo, pp. 215. London, 1821.

Erudition and talent, when confined to the individual, are nearly as useless to the community at large, as though they did not exist. They are like a river that takes a subterraneous course, and is known only to its own dark and solitary channels. But when they are made subservient to the general diffusion of knowledge, through the medium of the press, they are like a river winding beneath a fervid sun, through a thirsty country, whose branches irrigate the surrounding plains, permeate the arid soil, and scatter fertility over the face of nature. There is, moreover, in the intellectual, as well as in the physical, world, a moral obligation imposed on the favoured few, to extend a portion of the fruits of their endowments and acquirements to the indigent many —an extension which, in the medical profession particularly, is productive of the most beneficial consequences, not only to the profession itself, but to society at large.

The annals of medicine, ancient and modern, pregnant as they are with the most valuable information, are yet, from their vast extent, and the languages in which they are recorded, almost completely inaccessible to nine-tenths of medical practitioners. Hence it is, that the great mass of medical society enter on the actual duties of their profession with but a scanty stock of medical lore—namely, that which they have hastily noted down in the lecture room—picked up during the hurried movements of hospital practice—or purchased, on leaving the seats of education, in the shape of a few STOCK BOOKS, as they are not inaptly termed by the bibliopolists.

Now we are far from censuring this state of things, for really we know not how it can be otherwise under existing circumstances. But we are clearly of opinion that the man who, selecting some important class of diseases, lays open to us the stores of information, ancient and modern, collected or recorded on that particular subject—and that in a form and language accessible to all ranks of the profession, performs a great service to the rising generation—aye, and to no small proportion of those who consider themselves *melioris notæ* in the republic of medicine. This is the task which Dr. Cooke has undertaken, and his qualifications to execute it are acknowledged by all. His object appears to have been, to extract useful matter, on the subject of which he treats, from all the authors of reputation, of whatever age or country, and thus to communicate their sentiments in plain and perspicuous language. In the present publication our learned author has clothed the matter more in his own words, than in the former volume; and by so doing, he has endeavoured, in several instances, and that with success, to render diffuse passages succinct, and obscure parts of the subject more intelligible. Although Dr. Cooke's work is professedly a compilation, yet he has, on almost all occasions, as we shall shew in the sequel, introduced his own opinions and experience; and where he has not presumed to decide between jarring doctrines and modes of treatment, he has almost uniformly stated what he himself would be inclined to adopt or pursue, in such circumstances. The utility of this proceeding is so obvious that it is quite unnecessary for us to dilate upon it. The original communications which our author has received from his friends, particularly Dr. Philip, Mr. Charles Bell, Mr. Cooper, and others, must necessarily enhance the value of the publication; and with these few preliminary remarks we shall proceed to lay before our readers a brief analytical sketch of the work itself, which we doubt not will substantiate the justness of the preceding observations, and induce our readers to cultivate a much closer acquaintance with the original.

This *first* part of Dr. Cooke's second volume contains seven chapters and an appendix, viz. on the general *history* of palsy, hemiplegia, paraplegia, and paralysis partialis—on the *causes* of palsy—diagnosis and prognosis—and on the *treatment* of the disease. The appendix consists of an abstract from the minutes of the Army Medical Board, and an official return of cases of paralysis and apoplexy in the British forces, during a period of six months.

It is not a very easy task to present a connected view of so great a mass of excerpted opinions and observations, especially as the author has rendered the language terse and condensed by the method which he has adopted. Yet as many hundreds of our readers can never have access to the original, we shall endeavour to run a slender *line* of analytical delineation parallel with the great *chain* of the work itself, by which means a degree of communication between its parts may be preserved, and an *ex pede herculem* sample of the publication exhibited.

After detailing the *definitions* of ancient and modern authors, Dr. Cooke thinks, and we believe justly, that the following sentence may comprehend the chief characteristics of palsy.

"It is a disease in which there is a diminution, or an entire loss, of the power of voluntary motion, or of sensation, or of both, in some particular part or parts of the body, without coma." P. 3.

Our author remarks that he has generally found the precursors of palsy to be the same as of apoplexy; namely, vertigo, drowsiness, partial head-ache, numbness or torpor, loss of memory;—and more especially as forerunners of *palsy*, he has observed "a faltering inarticulate voice, drowsiness, a slight delirium, dimness of sight, or double vision, trembling, a numbness gradually ascending to the head, frequent yawning, weakness, distortion of the mouth, and disposition to faint."

"Palsy chiefly consists in the loss of the power of voluntary motion, for sensation in a greater or less degree generally remains; nay, in certain cases it is morbidly increased. I have seen several instances in which paralytic persons have felt very violent pain in the parts affected, particularly in the shoulder and arm. These remarks might be confirmed by quotations from various authors.—I never saw a case of palsy in which sensation was entirely lost; and an eminent physician of great experience asserts that a total loss of feeling in this disease is extremely rare." 5.

The vital and natural functions are generally but little affected in palsy. The opinion is very commonly received that the temperature of paralytic parts is below that of health.

Dr. Abercrombie is of different sentiments. He thinks, that the power of preserving a medium temperature is partially lost by paralytic limbs, so that they are more heated when exposed to heat, and more chilled when exposed to cold, than parts in a state of health. He illustrates this by the following fact:—

"A medical gentleman, on visiting a paralytic patient, was astonished to find the paralytic arm so intensely hot that he could not touch it. He was at first very much surprised; but found, upon enquiry, that the patient had, by the advice of a friend, applid to the arm a quantity of very hot bran, or something of that kind very hot, which had been removed a short time before his visit." 8.

Dr. Cooke remarks, that if this were generally the case, the temperature of paralytic parts ought to be always less than that of other parts, in these climates where the medium temperature of the air is far below human heat. Paralytic limbs often grow more soft and flaccid than natural, as well as wasted, and sometimes œdematous. Mr. Charles Bell informs our author that he has observed the nerves themselves, in such cases, lose much of their substance.

In this unhappy class of complaints, the energies of the mind are often much weakened—the memory, especially, is much impaired—sometimes partially so; curious examples of which are quoted from Galen and others.

"The passions of the mind are sometimes greatly affected by palsy. Persons naturally mild and placid, have become peevish and irritable under its influence; and sometimes a change of an opposite nature has been caused by it. I had several years ago an opportunity of seeing an illustration of this remark, in the case of a much respected friend. The person to whom I allude, had always, up to an advanced age, shown an irascible and irritable disposition; but after an attack of palsy, his temper became perfectly placid, and remained so until his death, about two years afterwards." 12.

In persons recovering from palsy, Dr. Cooke has often observed, that the parts most distant from the head are first restored. In hemiplegia it almost always happens that the leg recovers its power long before the arm. Our author adopts Dr. Cullen's nosological division of the disease into hemiplegia, paraplegia, and partialis.

I. *History of Hemiplegia.* This term does not occur in the writings of Hippocrates, Galen, or Aretæus. Paulus Ægineta appears to have been the first to designate this species of palsy. It is preceded, in a great proportion of cases, by apoplexy, but sometimes so slight as to escape notice. Dr. Cooke here brings forward a great number of hemiplegiac anomalies, if he may use the term, as well calculated to illustrate the history and nature of the disease. For these we must refer our readers to the work itself, from page 18 to 32.

"Hemiplegia sometimes terminates in a few days, but much more frequently the progress towards recovery is very slow. It often happens that an amendment to a considerable degree takes place, and then the disease remains stationary for months, or even for years: thus we frequently see persons, who have been attacked by hemiplegia, so far recovered as to be able to walk in an imperfect manner, one leg being dragged, with the foot turned out, without making any material further progress towards the healthy state." 33.

In some cases, however, the patient makes no improvement, is confined to bed, and dies gradually exhausted—"raro ad sanitatem superveniunt, et plerumque miserum spiritum trahunt, memoria quoque amissa." *Celsus*. The most distressing state, as Dr. Heberden observes, of this disorder, is that in which the powers of the mind are in the highest degree debilitated, and the passions become ungovernable, leading almost to madness, so that a person survives himself, as it were, and is reduced to a state of the utmost misery, if conscious of it—being unable to stand, to speak, to feed himself, or to retain the fæces or urine, yet continuing thus to live a burden to himself and all his friends.

II. *Paraplegia*. In this species of palsy, the superior parts of the body are seldom affected, unless the causes are seated in the upper parts of the spine. Galen appears to have been aware of this fact.

"If the spinal marrow, he says, be affected about the fifth vertebra, the hands will be wholly deprived of sense and motion; if about the sixth, the paralytic affection of them will be more partial, &c. and if below the eighth, they will not be affected at all." 37.

The paraplegia depending upon a diseased state of the spine and its connexions, is by far the most frequent. It is produced sometimes by mechanical injuries; but more generally it takes place gradually and constitutionally, especially in children.

"This disorder generally comes on slowly, and is preceded by langour, listlessness, and weakness in the knees. As it advances, a difficulty in properly directing the feet is experienced, and an involuntary crossing of the legs, with frequent tripping or stumbling are observable, and the legs or thighs are found to have lost a good deal of their sensibility, and to have become useless for the purposes of motion. When an adult is the patient, the progress of the distemper is much the same, but rather quicker." 39.

The paraplegia depending on an affection of the brain has been pointed out by Dr. Abercrombie, and more recently by Dr. Baillie, as we shewed in our last Number. Dr. Cooke has seen some cases where paralytic affections of the arms were occasioned by some complaint in the head; but does not recollect to have seen, in an adult, a loss of power in the lower extremities from that cause, and marked by the train of symptoms described by Dr. Baillie, whose accuracy of observation, however, is unquestionable. The anomalies of this species are very few, and only one is introduced, communicated by Dr. Hutchinson; but, dissection not being allowed, the case is imperfect.

III. *Paralysis Partialis* is that species which affects less than half the body—or some one particular part or organ. They may be considered as consisting in a want of sensation or of motion. Examples of the *first* we see in paralytic affections of the olfactory nerves, retina, gustatory nerves, auditory nerves, and nerves of touch:—of the *second*, in want of motion in the eye-lids, muscles of deglutition, organs of speech, urinary bladder, or in particular parts from the effects of lead. The vital organs are rarely affected in this way, being carefully guarded against such accidents, not only by the situation of the nerves of these organs, but by their own inherent powers.

After enumerating and exemplifying the various kinds of partial palsies, Dr. Cooke briefly notices the question often agitated by physiologists, how it happens that *motion* is sometimes lost, while *sensation* remains, and *vice versa*, if both functions are performed by the same nerves? The question puzzled Galen, as it had done Erasistratus and Herophilus long before him. It has since puzzled Forestus, Haller, Van Swieten, Sauvages, &c. Recent physiologists seem to have abandoned the hope of a solution. We shall introduce, however, the sentiments of two living authors of the present day.

"Dr. Wilson Philip, in compliance with my request, has given me his opinion upon it. Dr. Philip, who has carefully studied the nervous system, says, 'I think we must admit that the bundles of nerves, going directly from the brain or spinal marrow to any part of the body, contain nerves of two descriptions, one set adapted to convey the dictates of the will, the other to convey impressions from the part to the sensorium. This I think more probable, than that impressions move backwards and forwards in actually the same channels: one of these opinions must be correct. If the former is so, there is no difficulty in accounting for the feeling being lost, and the power of motion remaining, and *vice versâ*. Indeed these phenomena of disease seem to me to go some way towards proving the former opinion.

These observations may perhaps be applied to all nerves; but in other respects the ganglion nerves seem to obey laws very different from those which come directly from the brain and spinal marrow.'

"Mr. Charles Bell, whose physiological opinions deserve great attention, has also, in compliance with my desire, favoured me with his sentiments on this question. 'The nerves of sensation and motion,' he says, 'are bound together in the same membranes, for the convenience of distribution; but there is reason to conclude that they are distinct through their whole course, and as distinct in their origin in the brain, as in their final distribution to the skin and muscles; why then should we suppose that they are similarly affected in diseases of the brain. Even nerves of the same class, viz. the nerves of voluntary motion, are not affected in the same degree by disorder of the brain. When there is effusion upon the membranes of the brain, from excessive inebriety, or dropsical effusion of any kind, the muscles are influenced unequally; and it is remarkable that those muscles, and consequently those nerves, which are immediately under the influence of the will, are first affected or debilitated in the greatest degree, where there is a general disturbance of the brain. The slighter disorders affect the muscles of the eyes; in a greater degree, we shall see palsy of the face; in a still greater degree, we find the muscles of the limbs unequal to their office. The muscles of respiration are next affected; and the fibres of the hollow viscera retain their office whilst there is life. If nerves of voluntary motion are thus differently affected according to the distinction of their functions, we need not be surprised that nerves, in all respects so distinct as those of motion and sensation, should be differently affected by what appears to us a general and uniform affection of the brain." 58.

Dr. Cooke justly remarks upon these opinions that, until we can demonstrate the distinction between nerves of sense and nerves of motion (excepting in the eye and tongue where the distinction is evident) the question must be considered as involved in great obscurity, although the analogies above-mentioned are certainly greatly in favour of different sets of nerves for the two different functions.*

IV. *Etiology.* Some of the principal *predisposing* causes

* May not the function of a nerve depend upon the peculiarity of its structure where it *terminates* in an organ; as in the liver, where it regulates the secretion of bile; in the pancreas, the pancreatic secretion; in the kidney, the urine, &c.? We can discover no difference of structure in nerves going to different organs: one nerve sometimes distributing branches to organs of very different function. May not, therefore, the arrangement of the nervous filaments in the skin and other sensible parts, be peculiarly adapted for the function of sensation, without supposing the necessity of two different sets---one for motion, the other for feeling?—*Rev.*

of palsy, must be the same as those of apoplexy, enumerated and explained in Dr. Cooke's first volume, and treated of in this Series, *No.* I. *p.* 4, *et seq.* and the same may be said of the *exciting* causes. With the exception of paraplegia, paralytic affections, in general, occur in advanced age, especially in constitutions weakened by disease. Dr. Gordon's report from the Army Medical Board states, the chief exciting causes of apoplexy and palsy among the military to be, intoxication, exposure to the sun, drinking cold water, and bathing in the same—and that a predisposition to the disease is laid by long-continued attacks of fever, visceral diseases, epilepsy, and dysentery. The hemiplegic form being almost invariably preceded by an apoplectic fit, of greater or less duration or violence, " the causes, therefore, of both are the same."

Dr. Cooke details at considerable length the experiments of Dr. Serres, of whose pathological researches we gave a full account in our eclectic article on apoplexy. Of the *experiments*, however, we did not give an account, because we did not deem them quite conclusive, and we are glad to see that Dr. C. appears of a similar way of thinking. Indeed, he introduces a series of observations on this subject, which we are tempted to extract rather than abbreviate.

" The observations of M. Serres, and the facts which he has adduced, are highly worthy of our attention; and great credit ought to be given to him for his diligence in improving the peculiar opportunities for information which he enjoyed; but I think the conclusions which he has drawn from his reasoning are too general, and by no means strictly logical. He states, that he has, in many instances, thrown blood upon, and into the meninges, the cavities, and the substance of the brain, without producing apoplexy, or even somnolency; that upon the examination of a very great number of persons after death from apoplexy, he has found the meninges bearing evident marks of irritation (inflammation), accompanied with effusions of various kinds, and in various situations within the cranium; that sometimes he has observed the brain itself to have been injured in its substance, but without effusion; that in the former cases he has ascertained the preceding apoplexy to have been simple; and in the latter, combined with palsy; and that he has known many cases of apoplexy without effusion, and of effusion without apoplexy: and hence he concludes that he has overturned the doctrine of apoplexy and palsy from pressure, and has established a better system respecting the distinctions and nature of the diseases, than any hitherto presented to the world: but the accuracy of one of these conclusions, at least, may, I think, be reasonably doubted.

" If M. Serres chooses to denominate those apoplexies *meningeal* in which the meninges appear to have been diseased, accompanied with effusion, the brain being in a sound state, and those *cerebral* in which there is no effusion, but the brain itself is disordered; if M. Serres chooses to make this division of the disease, I see no objection: perhaps it is better than that of sanguineous and serous. But, though physiologists may approve of his distinctions, I much doubt whether they will be disposed to admit that he has overturned the doctrine of apoplexy and palsy from pressure.

"He has shown that blood has been effused in various situations within the cranium, without apoplexy; but he cannot hence fairly conclude that effusion never produces the disease. Since he admits that there is effusion in the meningeal apoplexy, how can he prove, even allowing it to be the consequence of what he calls irritation of the meninges, that the irritation, and not the effusion, is the immediate exciting cause of the disease. If compression by fluids were the cause of apoplexy, M. Serres says there could be no apoplexy without effusion; but the want of logical precision here is evident, unless we grant, what I believe few physiologists would admit, that compression from fluids is the *only* cause of the disease. That some degree of pressure may be made on the brain without producing either coma or apoplexy, may be conceded as proved by M. Serres's experiments; but it by no means follows that a different or greater pressure would not produce these diseases; indeed, both observation and experiment decidedly show, that compression does sometimes, according to its degree, give occasion, first to somnolency, and then to complete apoplexy. It is a fact perfectly well known, that after the operation of the trepan, pressure on the part deprived of cranium produces these effects: and innumerable instances might be adduced in which compression by depressed bone after accidents, has given occasion to coma and apoplexy.

"These observations are confirmed by direct experiments, which lead to positive conclusions; not to negative conclusions, like those of M. Serres. M. Portal treppanned the cranium of a dog; he compressed the dura mater and the brain, sometimes with his fingers, and sometimes with a bouchon of linen or of wood, and sometimes by water or mercury poured through the cavity made in the cranium. A funnel was adapted to the opening of the cranium, which was filled with water or mercury, so as to produce a graduated compression, more or less strong, upon the brain. In whatever way the experiment was made, it instantly produced the following effects—The animal ceased to bark; if the compression was increased, it became agitated by strong convulsions; if still further increased, a profound sleep took place, the convulsions ceased, and the respiration became stertorous. If the pressure was diminished, the breathing became more free, and the convulsions returned. This experiment was very often repeated by M. Portal, and always with the same results, except where the compression of the brain had been so strong, that its substance had been weighed down, *affaissée*.*

* Portal, Cours de Physiologie Experimentale, p. 248.

"Mr. Astley Cooper has been kind enough to give me an account of an experiment which he made upon a dog, with a view to ascertain what degree of pressure the brain could bear, which in part confirms M. Portal's experiment. He trepanned a dog, and detached the dura mater in a circle from the inner table of the skull, to the extent of half an inch. He then pressed upon the dura mater, so as to depress it about the fourth part of an inch, and the dog exhibited no signs of uneasiness. He then pressed upon it to half an inch of depression, and the animal showed great signs of uneasiness, endeavouring to escape from his grasp with all his efforts: he then pressed to three quarters of an inch, and the animal became torpid, breathed laboriously, and according to the declaration of Mr. Davie, who assisted him in the experiment, the pulse became slow and irregular. Suddenly he removed the pressure, and in half a minute the animal started from the table, turned several times round, as if giddy, and then staggered away." 88.

Keeping these observations in view, our very able author properly asks if, in a case of apoplexy, preceded by the usual symptoms, we should find, *post mortem*, a quantity of blood effused, without any other disease, (many instances of which are on record) would it not be reasonable to conclude that the effused blood first produced vertigo or somnolency, and afterwards, by its increase, a complete fit of apoplexy?—We would answer, yes. Our author thinks, perhaps justly too, (although against our own creed) that we err, both in attributing apoplexy *always* and *never* to pressure, as physiologists of opposite systems have done. Dr. Cooke has no doubt, however, "that in the generality of cases, apoplexy is produced by general, and palsy by partial compression." In this sentiment we fully coincide.

From hemiplegia, our author comes to paraplegia, and partial palsy. Paraplegia, he observes, in a great proportion of cases, is produced by morbid affections of the spine and its connexions, coming on slowly, and depending upon a constitutional disease. The cause is also sometimes seated in the head, as shewn by Dr. Baillie, Dr. Abercrombie, and others.

"A diseased state of the spine, and its connexions, commonly produces paralytic affections of the lower extremities; but if the injury be in the higher parts of the vertebral column, the muscles of the superior extremities sometimes are also affected, and even general palsy may be thus produced." 93.

The causes of partial palsies are numerous and various—sometimes seated in the head—sometimes the spine—sometimes, and Dr. C. thinks most frequently, in the nerves themselves. This disease occasionally depends on inflammatory action in the brain, or in the paralized parts, often accompanied with pain. The two following cases communicated by Dr. Abercrombie and Mr. Charles Bell, we shall insert here.

"Dr. Abercrombie, in his communication to me, describes a case of much paralytic disease from a circumscribed morbid cause in the head. 'A gentleman, aged 33, was seized with numbness and slight palsy of the left side, the numbness affecting also the left side of the face, the line being drawn with the utmost precision along the centre of the nose. He had no head-ach, and the pulse was natural. He was largely bled, and the affection went off in a few days; but from this time he was observed to be less acute in business, his memory was impaired, and he sometimes complained of his head. After three months, he was observed one day more confused and forgetful than usual, and in the night was seized with perfect apoplexy, which was fatal in twenty hours. On dissection, every part of the brain was found in the most healthy state, except that in the centre of the right corpus striatum there was an abscess, regularly and nicely defined, no bigger than would have contained a small pea. In the lower part of the left corpus striatum there was a more irregular suppuration of great fœtor extending over a space about the size of a small bean. There was no serous effusion, and no other morbid appearance of any kind.

"Mr. C. Bell has described to me a case of partial palsy, with great debility, preceded by inflammation of a nerve, accompanied with excruciating pain. In this instance, the inflammation was in the ulnar and fibular nerves, and the pain, which was of the most agonizing kind, had confined the patient for two years, and had quite subdued a powerful frame. He had in vain sought relief, says Mr. Bell, from some of our most distinguished friends, and he was left in a state of despair. Observing that the pain was confined to certain parts of the hands and feet, and that a dictinct class of muscles were become paralytic and shrunk, Mr. Bell's attention was directed to the corresponding nerves, and he found them tender, and acutely sensible to the slightest pressure. The accessions of pain were periodical and alternating, the feet being generally first attacked, and afterwards the hands. By repeated purging, and the application of leeches along the course of the nerves, together with other remedies to be described when I speak of the treatment of partial palsy, this patient was restored to health." 98.

Mr. Bell includes among the causes of partial palsy, long-continued exercise of particular muscles, or violence done to them—also irritation in the bowels, especially among the children of the poor. We saw a few years ago, an officer of rank, who always lost the power of speech, and sometimes of sight, whenever the gastric, biliary, and intestinal secretions were much disordered. We have repeatedly seen him become speechless in the middle of his dinner, when he could only make signs to his surgeon, who, on those occasions, gave him what this officer used afterwards facetiously to call the "doctor's thunderbolts," namely, brisk purgative pills of calomel and extract of colocynth, which invariably dissipated the paralytic affection, as soon as they began to operate.

Here our author notices the fumes or effluvia of metals, particularly lead, as well known causes of partial paralysis. Dr. Cooke was lately consulted by a gentleman who had a paralytic affection of one side of the face, without any assignable cause. On enquiry, however, it was found that he had slept two or three nights in a room where the bed was placed near a closet, the door of which had been recently painted, what is called a dead white. The external application of lead, even in its metallic form, has occasioned palsy.

"A person, a few years ago, was admitted into St. Bartholomew's Hospital, under the care of Dr. Powell, who was affected with palsy from this cause in a very extraordinary degree. The patient, who was a painter, was totally regardless of cleanliness in his business, and was in the habit of wiping his brush on the sleeves of his coat, so that he was particularly exposed to the fumes of the paint which he used. In this case, not only the hands of the patient were paralytic, but also the lower extremities, and the sphincter muscles of the bladder, so that he could not retain his urine." 104.

We must here pass over a great deal respecting the etiology of the disease under consideration, and also much interesting matter collected from various quarters respecting that curious phenomenon in palsy, namely, the opposite sites of cause and effect—the one being almost invariably seated in the right side when the other is in the left, and *vice versa*. Of all the explanations hitherto given, that of the *decussation of nerves in the brain*, particularly in the tuber annulare, though not satisfactorily demonstrated, appears to our author to be the most probable.

V. *Dissections*, *Diagnosis*, *Prognosis*. The *mort mortem* appearances in palsy very much resemble those after apoplexy, which have been described in Dr. Cooke's first volume, and also in the first No. of this series of our Journal. In this publication, however, Dr. Cooke has given a neat account of the pathological investigations of M. Serres, Riobé, Rochoux, and other late continental writers, with whom our readers are pretty familiar from the extracts we made in our article on apoplexy. The diagnosis, as our author remarks, is so clear that there can be no danger of confounding this disease with any other.

On the subject of PROGNOSIS there is much variety of opinion. The ancients, in general, considered palsies of long standing as incurable. Dr. Abercrombie, however, takes a more encouraging view of the subject, and thinks we have probably been too much in the habit of considering paralysis of long standing as depending on fixed and irremediable disease of the brain. Many cases, he observes, are on record, which lead to shake this opinion—some recovering very gradually, and, in a few weeks or months, leaving no trace of the disease. Dr. A. has not given us any marks by which we can discriminate these favourable from the more unfortunate cases. We fear that the following prognosis of our able author will prove too rigorously correct.

"As far as my own experience anables me to judge, the prognosis in the general palsies, must be almost always unfavourable. I have seen many cases of recovery from palsy in a very considerable degree; but I do not recollect more than one, or two cases, of a complete restoration, both of sensation and motion, in the whole of the side of a person who had been affected with a perfect hemiplegia. when this species of palsy depends upon an injury done to one side of the brain, which is almost always the case, I am inclined to think that the mischief is seldom, if ever, entirely obliterated, and the disease wholly removed. On the dissection of persons after palsy, either evident disease is found in the brain, or marks of the existence of former disease, which had given occasion to the complaint; and although Messrs. Rochoux and Riobé have adduced good reasons for believing that fluids effused have been absorbed, and that cavities in the brain have been sometimes closed, yet the mischief may not have been completely removed, nor the brain perfectly restored to its healthy state; and whilst any morbid cause capable of producing palsy continues in any degree to exist, it is natural to imagine that palsy in some degree would remain. Reasoning from appearances after death from palsy, would lead us to conclude that the disease almost always, in a greater or less degree, does remain. Instances may, no doubt, be adduced of perfect recovery from palsy; but I am persuaded that such are of very rare occurrence. If persons affected with hemiplegia do not become apoplectic in a short time, it often happens, that after a certain degree of amelioration, the disease becomes stationary, or very gradually proceeds, even for several years, before it terminates fatally." 130.

VI. *Treatment.* Our author enters first on the treatment of *hemiplegia*, the most common and the most important species of the disease. This species, as was before observed, being, in a great proportion of cases, the consequence of apoplexy, the means both of prevention and cure, must be nearly the same in both:—indeed they differ chiefly in degree. We shall pass over the preventive measures which are judiciously stated by Dr. Cooke at pages 131-2-3 of the work. On the actual accession of the disease, the measures must be nearly those employed in apoplectic seizures. Dr. Cullen was of opinion, that in hemiplegia of any standing there is probably either sanguineous or serous effusion. M. Serres almost always found a quantity of blood in cavities of the brain, produced by injuries done to that organ. Supposing then that pressure on the brain is the operating evil, the removal or diminution of that pressure is clearly our object;—and this can only be effectually attained by bloodletting, purgatives, emetics, diaphoretics, sialagogues, revellents, and discharges by means of blisters, issues, and setons. The ancients prescribed general and local bloodletting in this disease; and the moderns, who recommend the same measure in apoplexy, prescribe it in paralysis, but with caution. Those who doubt the propriety of bleeding in apoplexy, proscribe it wholly in palsy. Mr. Hunter was of opinion that in hemiplegia we ought to extract blood very largely, especially from the temporal arteries; and Dr. Abercrombie has published some cases illustrative of the success of this practice. The following are the sentiments of our author himself on this subject; and they are delivered with that cautious decision which marks the scientific practitioner.

"My own experience on this subject is in favour of the practice of blood-letting in hemiplegia, when accompanied with strongly marked apoplectic predisposition; and I do not recollect to have observed any mischief produced by it under such circumstances. I once, indeed, saw this species of palsy terminate in a fatal apoplexy soon after a free evacuation of blood, in the case of a gentleman seventy years of age, of a plethoric constitution, and free mode of living; yet I am convinced that bleeding, all circumstances considered, was in this case highly proper; nay, it is not very improbable I think, that by a still more copious evacuation of blood, the total abolition of sensation and motion might have been prevented. We have some cases on record, in which hæmorrhage from the nose, or from the hæmorrhoidal or menstrual vessels, have relieved persons affected by paralytic symptoms, especially when connected with the stoppage of the piles or the menses. With respect to the treatment of hemiplegia, as far as relates to the propriety of blood-letting, and the extent to which depletion by that means may be safely carried, it appears to me extremely difficult, if not impossible, with propriety to lay down any absolute general rules. Each individual case must be viewed in all its circumstances, and by a careful consideration of them, our practice should be regulated. Before we prescribe blood-letting in hemiplegia, we must investigate the age, strength, general constitution, and habits of the patient, and above all, the actual symptoms of the disease. In early, or even in somewhat advanced life, if plethora and the various symptoms formerly enumerated as tending to apoplexy were present, I should not scruple to bleed freely, both generally and topically. On the contrary, in great age, debilitated leucophlegmatic habit, dropsical tendency, &c. I should think it right to abstain altogether from this, and from every other powerful mode of depletion, unless there was an evident great determination of blood to the head, marked by flushing in the countenance, throbbing of the arteries, redness of the eyes, &c. In doubtful cases, indeed, the safest plan would be to evacuate blood only topically by leeches or cupping glasses, and to proceed as circumstances may direct, carefully watching from time to time the effects of the practice." 142.

There is much less caution necessary in the employment of purgatives in paralysis. "In all cases," says Dr. Cooke, "a free evacuation of the bowels is not only safe, but necessary." Boerhaave thought purgative medicines in certain cases, of more service than all others put together, provided the body could bear their power. *Van Sweeten* and Forestus are of nearly similar sentiments. Indeed all writers agree, as to the propriety of keeping the body open in hemiplegia; but that the cathartics should be suited to the nature of the case.

"The neutral salts and other purgatives called refrigerants, may be given where there is much determination of blood to the head, and in full habits; but in debilitated, leucophlegmatic, and dropsical cases, the more stimulating purgatives, such as aloes, calomel, scammony, colocynth, jalap, &c. may with more propriety be administered." 143.

Our continental brethren lay much stress on stimulating glysters; and emetics are prescribed with less scruple in palsy than in apoplexy by the practitioners of the present day—in France they are much employed. Dr. Cooke thinks, and we coincide with him, that in hemiplegia after apolexy, especially at the beginning, and in plethoric habits, emetics are doubtful remedies; but not so purgatives. Of diaphoretics, diuretics, and sialagogues, our author's experience does not enable him to speak. Indeed they can be but of very subordinate efficacy. These observations are meant to apply, of course, to hemiplegia in its early state, and more especially in its connexion with apoplexy. But when the disease has subsisted for a length of time, and the apoplectic symptoms have disappeared—when determination of blood to the head is no longer apparent—then the *methodus medendi* becomes considerably modified, and a stimulant class of remedies may be safely ventured on, though previously inadmissible. The chief external stimulants are frictions, blisters, sinapisms, fomentations, balnea calida, electricity, and galvanism. The internal are volatile salts, acrid vegetables, aromatics, essential oils, and resinous substances. The ancients, Dr. Cooke remarks, were much too free in the employment of stimulants in palsy, from always viewing the disease as one of debility, coldness, and obstruction. Dr. Cullen made some useful distinctions in this respect. The excitement of the passions has been resorted to, in former times, as a stimulant in palsy; and some curious instances of their efficacy are on record. But, as Dr. Cooke has justly observed, these instruments are not sufficiently under our control and management, to ever become of much practical advantage.

Of the external stimulants, our author has derived most benefit from friction by the hand or flesh-brush, which may be rendered more efficacious by stimulating liniments, as the fossil acids and volatile alkalies, combined with oil or lard—essential oils, &c. Blisters and sinapisms are very generally employed in palsy—yet they should be used with caution, when sensation and motion are nearly extinguished.

"Some practitioners are in the habit of applying blisters, or other stimulants to the head, immediately after the accession of hemiplegia; but I am of opinion, that we ought not to make such applications in plethoric constitutions, and especially when the disease is the consequence of apoplexy, till some blood has been taken away; and I think, that when such stimulants are used, they should be applied on that side of the head which is opposite to the paralytic side; because anatomists have ascertained, as above mentioned, that in a very great proportion of instances, the cause of hemiplegia is seated in some part of the brain, opposite to the side affected." 150.

The thermal waters, especially those of Bath and Buxton, have been greatly extolled, and doubtless they have deserved, like most other remedies, a modicum of the praise lavished on them. Sudden *cold* applications have also had their partizans. Dr. Cooke is inclined to think most favourably, upon the whole, of the warm applications, as most under our command. "If cold does not produce re-action; or if it give occasion to a very great re-action, it would probably do mischief." Electricity has been very generally employed in hemiplegia, especially on the Continent, and they say with success:—nevertheless, it seems, of late years, to have fallen into disrepute. There are many histories on record, where it has been unequivocally injurious. It is no doubt a powerful excitant of the nervous system. But what becomes of the vascular system the while? Is not paralysis very generally—indeed, most commonly, the effect of pressure on the brain or spinal marrow: what success then are we to expect from electricity? We give our author's sentiments in his own words.

"From my own observations of the effects of electricity in paralytic affections, I venture to recommend a trial of it under the circumstances, and with the cautions pointed out. I have in many cases seen it materially useful, and I do not recollect a single instance in which it appeared to do mischief. Although Dr. Cullen, as above-mentioned, places more dependence upon a repetition of electricity, than upon its force, I wish to observe that, from experience, it appears to me to be chiefly serviceable on its first application, and that its beneficial effects are by no means proportioned to the length of time of the employment of it. Dr. Franklin observed, 'that the paralytic persons whom he electrified were generally restored for a few days in the beginning, but that they afterwards either did not mend, or that they relapsed into their former state.'

I saw, some years ago, a case of complete paraplegia, in which the good effects of electricity were at first extraordinarily great, but they were not permanent; for though the application of this power was for a considerable time continued, the patient experienced a diminution, rather than an increase, of sensation and motion, in the parts affected." 159.

Mr. Partington, whose experience is considerable, and on whose judgment we certainly should be much inclined to rely, considers electricity as superior to galvanism, especially in its application to the head and neighbouring parts.

The actual cautery is said to have been successfully employed by the Egyptians in apoplexy, palsy, and epilepsy; and the moxa is much used on the Continent. Internally, the rhus toxicodendron, nux vomica, horse-radish root, and the seeds of mustard, have been often administered, with various success.

Half a grainof the toxicodendron (powdered leaf) may be given thrice a day; and the quantity gradually increased to two, three, or four grains, watching carefully the effects. It commonly produces a twitching or convulsive motion, or a sense of tingling or pricking in the paralytic part; and in these cases its efficacy is most conspicuous. Dr. Alderson, of Hull, has published several striking cases of the utility of this remedy in paralysis which afford encouragement, if there be any faith in medicine, to a further trial of it in those cases where the employment of stimulants is not contra-indicated.

Of late years, the nux vomica, a medicine a good deal resembling the rhus toxicodendron in its effects on the constitution, has been much employed, especially on the Continent. Dr. Fonquier has been in the habit of giving it in the form of powder and extract, beginning with small doses, and gradually increasing them as far as fifty grains in a day.

"In a short time after it has been taken, spasmodic contractions of the muscles, he says, in a greater or less degree, or more or less general, are produced. Among the unpleasant effects of the nux vomica, especially in large doses, delirium, a sense of heat and oppression, and anxiety, may be mentioned. Sometimes general tetanus, with loss of speech and the power of deglutition, accompanied with difficulty of respiration, pulsation of the heart, and dysuria, have been experienced; whilst the good effects of this remedy are manifested by a gradual return of power in the paralytic parts." 168.

Dr. Dickson, of Clifton, has communicated the results of two cases, in which this able physician tried the nux vomica in hemiplegia with some advantage; and Mr. Rose, of Swafham, has published two cases, in which the powerful, rather than the beneficial, effects were sufficiently conspicuous. In the eighth volume of the Medical Repository, Dr. Granville has communicated the result of Professor Dumeril's experience of this medicine in a case of palsy, where complete success was obtained. Many cases, on the Continent, have since occurred, where the nux vomica appears to have produced much benefit in this deplorable malady; and we sincerely hope it may continue to support its character. Cantharides, administered internally, have been productive of some advantage in Dr. Cooke's hands; but it must be given with caution.

In the treatment of paraplegia, where the cause appears to be in the spine, issues applied on each side of the vertebral column will, of course, be the principal measure to be depended on.

"In cases," says Dr. Cooke, "of paraplegia from diseased spine in scrofulous habits, I am of opinion that considerable advantage has in many cases been derived from a proper attention to air, exercise, diet, friction, sea-bathing, mercury in alterative doses, and certain tonic medicines, especially the bark." 184.

Dr. Baillie's remarks on this species of the disease have been fully given in our last number, page 391 *et seq.* to which we refer.

Of the partial palsies, *amaurosis* is the most important: for the treatment of which, we must refer to the volume itself, and to our review of Dr. Vetch's work in this, and Mr. Travers' volume in our next number. With the following, we must conclude our extracts from Dr. Cooke's publication.

"In those cases, in which the loss of motion of particular parts is accompanied with pain, Mr. Bell advises the application of leeches along the course of the nerves of the part affected, a free administration of cathartics, friction with laudanum, and proper supporting bandages. This plan, with the addition of opium and colchicum, internally taken in considerable doses, proved effectual in relieving the distressing case above-mentioned under the care of Mr. Bell." 207.

Towards the end of the volume, Dr. Cooke takes notice of the *paralysis agitans*, or shaking palsy, described by Dr. Parkinson; an exquisite specimen of which we saw a few years ago at Gosport; the unhappy victim of which is still probably alive. Mr. Parkinson's treatment of this remarkable variety, consists in local detractions of blood from the upper part of the neck; after which, blisters are to be applied to the same part, and a purulent discharge maintained by means of savine cerate. If this fail, large issues are to be established on each side of the vertebral column, near the cervical region, and kept open, with proper attention to the state of the bowels.

If we have been successful in rendering such an account of Dr. Cooke's work to the public, as may enable them to judge for themselves respecting its nature, object, and execution, we have done all that we attempted, and all indeed that the public require. When the scales of Justice are thus placed in hands, "*quas nulla movent vota precesve*"—the *opinions* of critics are but—" as dust in the balance."

